# Some Points in the Application of Hydrochlorate of Cocaine to Dental Surgery

**Published:** 1885-01

**Authors:** N. J. Hepburn

**Affiliations:** Ophthalmic and Aural Surgeon to the Demilt Dispensary, Assistant Surgeon to the Manhattan Eye and Ear Hospital, District Physician to the New York Lying-in Asylum, Clinical Assistant at the College of Physicians and Surgeons, etc.


### SOME POINTS IN THE APPLICATION OF HYDROCHLORATE OF COCAINE TO DENTAL SURGERY.

BY N. J. HEPBURN, M. D.,

*Ophthalmic and Aural Surgeon to the Demilt Dispensary, Assistant Surgeon to the Manhattan Eye and Ear Hospital, District Physician to the New York Lying-in Asylum, Clinical Assistant at the College of Physicians and Surgeons, etc.*

The main obstacle to the general adoption of the new anæsthetic has seemed to be the uncertainty in regard to the best mode of application, or the difficulty in reaching the part which the operator desires to affect. This is especially the case in dentistry, and it is in the hope of assisting to open the way for a fair trial of the alkaloid and its salts in dental surgery that I venture the following remarks.

Erythroxylon Coca and its alkaloid have been so exhaustively written up in the medical journals of this city during the past two months, that we may pass over the natural history and constitution of the plant, as well as its triumphs in ophthalmic surgery, and confine our attention to a few facts which have been obtained by actual experiment.

A four per cent. solution of cocaine hydrochlorate in water was dropped by Dr. G. W. Weld, of New York, into the cavity of a tooth whose pulp he wished to remove; in a few minutes this was extracted without pain being felt by the patient.

Having observed in several operations about the face that the anæsthesia produced by cocaine was apparently confined to the tissues supplied by the nerve over which it had been used, it occurred to me to ascertain whether anæsthesia of a part might not be produced by application of the anæsthetic to the trunk of the nerve of sensation distributed to that part. Experiments were accordingly made, by injecting varying quantities of the solution over the supra-orbital, infra-orbital, mental, and dental nerves, in each case endeavoring to have the needle of the syringe approach as closely as possible the nerve to be acted upon. The results of these experiments only served to confirm previous impressions, and we are now enabled to state positively that the anæsthetic applied to a nerve of sensation will produce insensibility of the tissues to which that nerve is distributed, beyond the point of application. For example:

Eight drops of a four per cent. solution were injected over the supra-orbital nerve at its foramen, and in two minutes there was complete anæsthesia of that part of the forehead and scalp to which the nerve was distributed, lasting twenty minutes. A similar application was made over the infra-orbital foramen on the right side, using ten drops of the solution. In five minutes there was complete anæsthesia of the cheek of the same side, of the teeth in the right upper jaw, from the median line to the first molar, and partial anæsthesia of one side of the nose, as well as of the right ear and vicinity. The anæsthesia lasted twenty minutes, and sensation was imperfect for a much longer time. While sensation was abolished the teeth were repeatedly struck, pieces of wedge inserted between them, the gums pierced with pins and pinched with forceps, without producing discomfort on the part of the patient.

Another experiment was made on the same patient, two days later, when the syringe needle was inserted through the buccal mucous membrane, in close proximity to the entrance of the inferior dental nerve of the left side into the dental foramen. The result was anæsthesia of the lower lip, molar, canine and incisor teeth, and partial anæsthesia of the tongue on that side. It appeared possible to extract any of the teeth in that region without pain, but as the patient objected to this sacrifice in the cause of science, it was not attempted. The anæsthesia lasted just one half-hour from the time of administration.

These experiments were repeated several times, on different individuals, between the 11th and 28th of November, and with slight variations produced substantially the results above recorded. In each case sensation was measured by the Carroll æsthiometer.

Since these observations were made, there has appeared in the *New York Medical Journal*, of December 6th, 1884, a letter from Dr. R. J. Hall, giving a very clear and complete description of experiments made by himself in the same direction, which will well repay perusal. Numerous applications have been made to aching cavities at the Demilt Dispensary, with satisfactory results.

The use of twenty to thirty drops of a four per cent. solution will often be followed by constitutional effects which, while not dangerous, are somewhat disagreeable to the patient. These are, increase in the pulse and respiration, some general anæsthesia, staggering gait, double vision, dilated pupils, dizziness, hallucinations, and one or two observers have reported nausea. These effects pass off in an hour or two.

A case is on record where five grains of cocaine were taken at one dose, without fatal effect. It is, however, advisable to use not more than one-half to three-quarters of a grain at a sitting, if it is desired to avoid the unpleasant sensations referred to above, and less than one-half grain will produce sufficient anæsthesia for short operations.

Since the above was written, a case has been brought to my notice of the painless extraction of an incisor under cocaine, but the particulars have not been furnished, so that the account is not complete.

A fact worth bearing in mind in this connection is that the use of cocaine diminishes congestion, blanches the part, and to some extent lessens hemorrhage.

